# Trends in ‘Watch’ and ‘Reserve’ Antibiotic Use in Primary Care in Kazakhstan: The Imperative for Enhancing Stewardship Strategies

**DOI:** 10.3390/antibiotics14100963

**Published:** 2025-09-25

**Authors:** Kamila Akhmetova, Larissa Makalkina, Lyudmila Pivina, Lisa Lim, Nurlan Aukenov, Kuandyk Boranbayev, Rimantas Stukas, Tatiana Belikhina, Nurgul Aldiyarova, Assiya Turgambayeva, Yuliya Semenova

**Affiliations:** 1 Department of Public Health and Management, Astana Medical University, Astana 010000, Kazakhstan; akhmetova.km@amu.kz (K.A.); turgambaeva.a@amu.kz (A.T.); 2 Department of Clinical Pharmacology, Astana Medical University, Astana 010000, Kazakhstan; makalkina.l@amu.kz (L.M.); nurgul.aldiyarova@gmail.com (N.A.); 3 Department of Emergency Medicine, Semey Medical University, Semey 071407, Kazakhstan; lyudmila.pivina@smu.edu.kz; 4 Graduate School of Public Policy, Nazarbayev University, Astana 010000, Kazakhstan; lisa.lim@nu.edu.kz; 5 Research Administration, South Kazakhstan Medical Academy, Shymkent 160001, Kazakhstan; n.aukenov@skma.edu.kz; 6 School of Medicine, Nazarbayev University, Astana 010000, Kazakhstan; kuandyk.boranbayev@nu.edu.kz; 7 Faculty of Medicine, Vilnius University, 01513 Vilnius, Lithuania; rimantas.stukas@mf.vu.lt; 8 Department of Scientific Management, National Research Oncology Center, Astana 020000, Kazakhstan

**Keywords:** antimicrobial resistance, antibiotic consumption, antimicrobial stewardship, defined daily doses, primary care, healthcare providers, Kazakhstan

## Abstract

**Background/Objectives**: This study aimed to analyze the consumption of systemic antibacterials (WHO ATC J01) in primary care in Kazakhstan from 2017 to 2024 and to estimate the potential contribution of different primary care providers to the use of “Watch” and “Reserve” antibiotics. **Methods**: The Global Antimicrobial Resistance and Use Surveillance System methodology was applied to calculate defined daily doses per 1000 inhabitants per day (DID) for ATC J01 overall, as well as at ATC levels 3 and 5. Forecast modeling was performed using ARIMA (0,1,0), ARIMA (0,0,0), and Simple models to project trends through 2030. Pearson’s correlation was computed for each category of primary healthcare provider and each chemical agent belonging to the “Watch” and “Reserve” groups. **Results**: Antibiotic consumption increased by approximately 1.5 times in 2024 after remaining relatively stable from 2017 to 2023. The forecasts suggest that the share of “Access” group antibiotics will continue to decline, reaching 33.71% by 2030, whereas the share of “Watch” group antibiotics is expected to increase proportionally, reaching 65.73% by 2030. The per capita densities of primary care providers correlate with the consumption of certain “Watch” and “Reserve” group antibiotics. **Conclusions**: This study demonstrates that the primary care sector in Kazakhstan is characterized by a disproportionately high and increasing use of “Watch” antibiotics, a rising trend in “Reserve” antibiotic consumption, and a declining share of “Access” antibiotics. These findings highlight a need to prioritize stewardship interventions that target the reduction in “Watch” and “Reserve” antibiotics, while promoting the use of “Access” antibiotics.

## 1. Introduction

Antimicrobial resistance (AMR) is a global health challenge that continues to escalate, with recent forecasts of 1.91 million deaths annually attributable to AMR and 8.22 million deaths associated with AMR by 2050 [1]. Multiple factors contribute to this anticipated growth, including both socioeconomic and healthcare-related determinants. Among these, the unavailability, inaccessibility, and irrational use of antibiotics play the most critical roles [2]. The development of novel antibiotics—particularly those targeting Gram-negative pathogens—together with the implementation of antimicrobial stewardship (AMS) programs to promote the prudent use of existing antibiotics could mitigate this scenario and reduce the global burden of AMR [3].

Monitoring antibiotic consumption forms the foundation of an effective AMS program, as it provides baseline data on consumption patterns and enables the evaluation of stewardship interventions over time [4]. High-income countries have established robust surveillance systems, with the European Centre for Disease Prevention and Control (ECDC) serving as a leading example [5]. The World Health Organization (WHO) Regional Office for Europe [6] and the WHO Regional Office for the Western Pacific [7] also regularly collect and report data on antibiotic consumption in their member states. However, comprehensive monitoring remains lacking in many parts of the world, with data being particularly scarce from low- and middle-income countries (LMICs) [8].

In recent years, diverse international policies have been deployed to manage antibiotic consumption in primary care. For example, in England, the national programs “TARGET and Start Smart Then Focus” provide toolkits for AMS interventions; however, while many clinical commissioning groups and acute trusts have formally or informally reviewed these toolkits, far fewer have developed dedicated action plans or embedded roles for antimicrobial pharmacists, especially in primary care [9]. In Italy’s Veneto Region, the ARCO Project highlights governance models integrating both hospital and community settings, revealing variability in adherence, resource allocation, training, and infection prevention and control across sectors [10]. In the Eastern Mediterranean Region, efforts are underway to integrate One Health approaches and stewardship policies amid challenges such as fractured health systems, limited surveillance, and inconsistent regulation, but the outcomes and implementation remain heterogeneous across countries [11].

In general, primary care remains relatively understudied in terms of antibiotic consumption in LMICs, where antimicrobial stewardship initiatives are often limited or inconsistently implemented [12]. Regulatory oversight of over-the-counter (OTC) antibiotic sales is frequently weak, and primary care providers—including general practitioners (GPs), community internists, and pediatricians—often lack access to or adherence to evidence-based prescribing guidelines [13]. Moreover, the primary care sector accounts for a significant proportion of total antibiotic consumption in many countries worldwide, a trend reinforced by the growing emphasis on reducing unnecessary hospital utilization [14]. Inappropriate antibiotic use in primary care not only undermines treatment outcomes but also contributes to the spread of AMR, which subsequently poses challenges in hospital settings [15].

The Republic of Kazakhstan (hereafter referred to as “Kazakhstan”) is an LMIC in Central Asia. Following its independence in 1991 after the breakdown of the Union of Soviet Socialist Republics, the country inherited a Soviet-style healthcare system. At the primary care level, healthcare is organized around a network of GP clinics and polyclinics staffed by specialist physicians [16]. Kazakhstan has made efforts to introduce AMS initiatives, including regulations prohibiting OTC sales of antibiotics. However, regulatory enforcement has weakened since the COVID-19 pandemic [17]. A recent nationwide evaluation of antibiotic consumption revealed a predominance of “Watch” group antibiotics according to the WHO AWaRe classification [18]. Nevertheless, no comprehensive evaluation has been conducted to specifically assess antibiotic consumption in primary care, including trends and categories according to the AWaRe classification. This study addresses this gap by analyzing eight-year trends (2017–2024) in primary care antibiotic consumption by pharmacological subgroup (Anatomical Therapeutic Chemical (ATC) classification system level 3 (ATC3)), chemical substance (ATC5), AWaRe category, and route of administration. In addition, this study provides projections for antibiotic consumption under a “no action” scenario until 2030. A secondary aim is to estimate the potential contribution of different primary care providers, including GPs, community internists and pediatricians, as well as pharmacists, to the use of “Watch” and “Reserve” antibiotics. The findings of this study are expected to inform strategies for strengthening Kazakhstan’s national AMS program.

## 2. Results

### 2.1. Antibiotic Consumption in the Primary Care, 2017–2024

Table S1 presents a detailed breakdown of antibiotic consumption in the primary care from 2017 to 2024, disaggregated by ATC3 and ATC5 codes. The overall consumption, measured in defined daily doses per 1000 inhabitants per day (DID), ranged between 8.50 DID (observed in 2021) and 13.43 DID (observed in 2024). The average annual percentage change (AAPC) for total primary care antibiotic consumption was 2.58 (95% CI: −2.96–8.43). The most consumed pharmacological classes were beta-lactam antibacterials, penicillins (J01C), other beta-lactam antibacterials (J01D), macrolides, lincosamides, and streptogramins (J01F), and quinolone antibacterials (J01M). Together, these classes accounted for more than 70% of total consumption. In contrast, combinations of antibacterials (J01R) represented the least consumed pharmacological class, contributing to less than 1% of total use. The most substantial growth was observed for macrolides, lincosamides, and streptogramins (J01F), with an AAPC of 11.01 (95% CI: 4.96 to 17.42), followed by other beta-lactam antibacterials (J01D) with an AAPC of 4.77 (95% CI: −1.55 to 11.50) and quinolone antibacterials (J01M) with an AAPC of 3.52 (95% CI: −1.48 to 8.78). In contrast, aminoglycoside antibacterials (J01G) exhibited the steepest decline, with an AAPC of −27.24 (95% CI: −40.83 to −10.52).

Table 1 presents the rates of antibiotic consumption from 2017 to 2024 by pharmacological group expressed as DIDs. Cephalosporins (J01DB, J01DC, J01DD, J01DE, and J01DI) constituted the largest share of total consumption, with 2.81 DIDs consumed in 2024 (20.9%). The second and third most consumed groups of antibiotics were aminoglycosides (J01GA and J01GB) and fluoroquinolones (J01MA), respectively. However, the most significant growth in consumption rates was observed for carbapenems, with an AAPC of 37.29 (95% CI: −2.88–94.06), and polymyxins (J01XB), with an AAPC of 30.75 (95% CI: 5.00–62.82). The latter trend is alarming given that both representatives of this group available in Kazakhstan (colistin and polymyxin B) belong to the “Reserve” group on the basis of the AWaRe classification.

Overall, 23 antibiotics accounted for 95% of the antibiotics consumed in the primary care. The most frequently consumed antibiotics were azithromycin and ciprofloxacin, together comprising 20–25% of the total antibiotic use; both belong to the “Watch” category. Between 2017 and 2019, azithromycin ranked 4th–6th in consumption, but its use peaked in 2020, and from then until 2024, it consistently remained among the top-ranked antibiotics. The third most consumed antibiotic was amoxicillin, an “Access” group antibiotic, while its combination with clavulanic acid ranked fifth. Overall, antibiotics from the “Watch” group predominated in the structure of primary care antibiotic consumption (Table 2).

**Figure 1:**
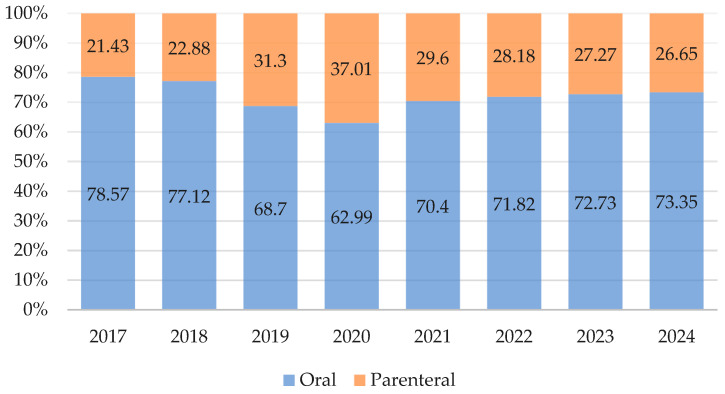
Antibiotic consumption by route of administration, 2017–2024 (%).

The most common route of antibiotic administration is oral, although the parenteral route still accounts for a substantial proportion of antibiotic consumption despite being in the primary care sector. The lowest proportion of parenteral consumption was observed in 2017 (21.43%), whereas the highest was recorded in 2020 (37.01%). Overall, the trend in oral administration decreased, with an AAPC of –0.63 (95% CI: –3.32–2.13), whereas the trend in parenteral administration increased, with an AAPC of 2.25 (95% CI: –4.35–9.29) (Figure 1). The unexpectedly high proportion of parenteral antibiotic use in primary care may indicate irrational prescribing practices and overuse of injections.

**Figure 2:**
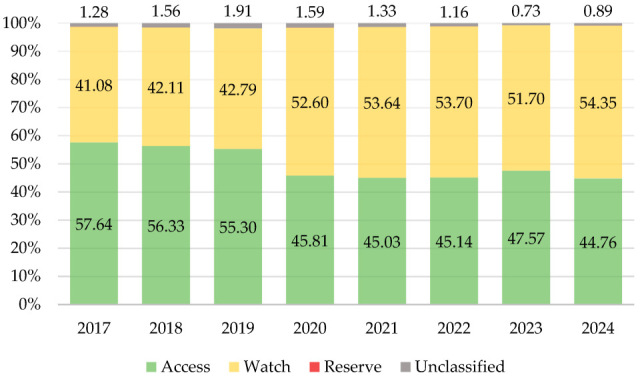
Antibiotic consumption by the categories of AWaRe classification, 2017–2024 (%).

Figure 2 presents an overview of antibiotic consumption based on the AWaRe classification during the study period. The consumption of “Access” group antibiotics ranged between 57.6% (2017) and 44.8% (2024), with an AAPC of −3.79 (95% CI −6.09–−1.42). The consumption of “Watch” group antibiotics experienced proportional growth from 41.1% in 2017 to 54.4% in 2024, with an AAPC of 4.49 (95% CI 1.91–7.13). The consumption of the “Reserve” group and unclassified antibiotics accounted for consistently small shares (together < 2%). “Reserve” group antibiotic consumption showed a positive trend (AAPC 15.35, 95% CI: −23.31–73.50), whereas unclassified antibiotics showed a declining trend (AAPC −6.77, 95% CI: −15.19–2.48). The decline in “Access” antibiotics and the rise in “Watch” antibiotics indicate a shift toward broader-spectrum agents, which is concerning for AMR development.

Table 3 presents the projected rates of antibiotic consumption by AWaRe categories until 2030, assuming that the observed trends remain unchanged. The forecasts suggest that the share of “Access” group antibiotics will continue to decline, reaching 33.71% by 2030, whereas the share of “Watch” group antibiotics is expected to increase proportionally, reaching 65.73% by 2030. Moreover, the consumption of “Reserve” and “unclassified” group antibiotics is projected to remain largely stable.

### 2.2. Antibiotic Consumption and Primary Care Provider Rates, 2017–2024

Table 4 presents the results of the correlation analysis between the consumption of “Watch” and “Reserve” group antibiotics, expressed as DID, and primary care provider rates per 1000 people. A strong, positive, and statistically significant correlation was observed between the rate of GPs and the consumption of cefpodoxime and moxifloxacin. Conversely, a strong, negative, and statistically significant correlation was found between GP rates and the consumption of erythromycin, roxithromycin, streptomycin, kanamycin, pefloxacin, and pipemidic acid. In contrast, the rates of community internists and pediatricians strongly positively correlated with the consumption of erythromycin, spiramycin, midecamycin, roxithromycin, josamycin, streptomycin, pefloxacin, and pipemidic acid but strongly negatively correlated with the consumption of cefpodoxime, moxifloxacin, and colistin. With respect to community pharmacists, a strong negative correlation was observed between their per capita rates and the consumption of ceftaroline-fosamil and vancomycin.

## 3. Discussion

The findings of this study provide evidence relevant to strengthening the national AMS program. First, it is among the few investigations to report patterns and eight-year trends of antibiotic consumption in the primary care sector of an LMIC during the past decade. Second, it explores potential associations between the consumption of “Watch” and “Reserve” group antibiotics and the per capita density of primary care providers over the same period. These findings need to be discussed in more detail.

### 3.1. Antibiotic Consumption in the Primary Care Sector

This study revealed that after remaining relatively stable from 2017 to 2023, when antibiotic consumption in the primary care sector ranged between 8.33 DID in 2022 and 9.41 DID in 2017, consumption increased by approximately 1.5 times in 2024. The exact reason for this growth is not known. Globally, such increases are often associated with the emergence of novel infectious diseases or outbreaks of existing pathogens [19], which does not apply to Kazakhstan, as no major infectious disease outbreaks were reported after the COVID-19 pandemic. Therefore, a more plausible explanation may lie in the suboptimal implementation of the national AMS program. Kazakhstan has established a national AMS framework that includes a set of measures designed to promote the rational use of antibiotics, such as awareness-raising campaigns targeting the general population, as well as research on antibiotic use and AMR. Among the core components of this framework is the prohibition of OTC antibiotic sales [20].

The prohibition of OTC antibiotic sales was enforced through inspections of community pharmacies and monitoring of antibiotic procurement, with pharmacies selling antibiotics OTC subject to fines [21]. However, during the acute surge of COVID-19 in the summer of 2020, community pharmacies faced shortages of essential medicines, which led to a relaxation in the enforcement of this legislation [22]. It may be hypothesized that after the pandemic, enforcement levels did not fully return to their prepandemic state, potentially contributing to the observed increase in antibiotic consumption in primary care [17]. Notably, electronic prescription systems have not yet been implemented in Kazakhstan, largely because several parallel healthcare software platforms that lack integration exist [23]. Wider adoption of electronic prescriptions could help curb OTC antibiotic sales in the country [24]. However, these assumptions require empirical validation, as evidence from Kazakhstan is lacking.

Another important problem identified by this study is the predominance of “Watch” group antibiotics in the structure of antibiotic consumption, which increased from 41.1% to 54.4% during the study period and is projected to reach 65.7% by 2030. The WHO introduced the AWaRe classification in 2015 to guide antibiotic stewardship and, in 2019, set a global target in which at least 60% of total antibiotic consumption should come from the “Access” group [25]. By implication, the combined consumption of the “Watch” and “Reserve” groups should not exceed 40%. In 2024, the WHO updated this goal, calling on all member states to ensure that, by 2030, “Access” group antibiotics constitute at least 70% of total use [26]. If current trends in Kazakhstan persist, the country will not meet this target, as projections indicate that “Access” group consumption may decline to 33.7% by 2030.

The predominance of “Watch” group consumption should be addressed through targeted AMS interventions, which, to be effective, must address the underlying reasons for this trend. One reason is the limited availability of “Access” group antibiotics in Kazakhstan, as not all WHO-recommended medicines listed in the Essential Medicines List Antibiotic Book [27] are registered in the country [28]. Another reason is that healthcare provision in Kazakhstan is regulated by national standards of care [29], many of which are outdated and were developed without reference to WHO AWaRe recommendations [22]. Therefore, the planned update of these national standards should place particular emphasis on aligning antibiotic selection with WHO guidance and the best available international evidence. In addition, the Ministry of Health (MoH) should implement measures to expand the registration and availability of “Access” group antibiotics in Kazakhstan.

The fact that azithromycin and ciprofloxacin, both from the “Watch” group, are the two most frequently consumed antibiotics in primary care in Kazakhstan, together accounting for more than 25% of all antibiotic consumption, further illustrates this trend. During the prepandemic period (2017–2019), azithromycin ranked 4th to 6th among all antibiotics consumed. Its use peaked in 2020 and has remained high through the present. This surge is likely attributable to early studies suggesting potential antiviral properties of azithromycin during the initial stages of the COVID-19 pandemic [30], although these claims were not supported by subsequent research [31]. An earlier study on azithromycin use in Kazakhstan also noted that it is frequently recommended in national clinical standards of care for a wide range of infections and inflammatory conditions [22]. Ciprofloxacin consumption consistently ranked 2nd during the study period. This antibiotic is also widely recommended in the national standards of care [29], highlighting the need to revise these standards, given the well-documented association of ciprofloxacin with the development of AMR [32].

This study revealed that the proportion of antibiotics administered parenterally in primary care is unusually high, ranging between 21.4% and 31%. Globally, outpatient parenteral use is typically much lower, with comparable rates in Europe generally observed only in the hospital sector [33]. In Kazakhstan, the tradition of administering medicines parenterally is a legacy of the Soviet healthcare system and remains deeply ingrained in both patients and physicians, who often believe that the parenteral route is more effective than oral administration [34]. However, this belief is not supported by the current best evidence, which demonstrates that oral antibiotic administration is equally effective in most cases and is associated with greater safety [35]. Therefore, the AMS program could be strengthened with this message, particularly through information campaigns targeting both patients and healthcare providers. Furthermore, research into the barriers to adopting oral formulations in Kazakhstan’s outpatient settings is warranted to inform effective policy and practice interventions.

### 3.2. Antibiotic Consumption and Primary Care Providers

The national AMR framework envisages the development of clinical practice guidelines on the rational use of antibiotics by 2025–2026 [20]. Such guidelines are urgently needed for the Kazakhstani medical community, alongside planned dissemination efforts specifically targeting primary care providers, who constitute the bulk of the country’s human healthcare workforce [36]. These guidelines should be aligned with the WHO AWaRe classification [37] and prioritize the use of “Access” group antibiotics at the primary care level.

Physicians are key drivers of antibiotic prescribing, and even in countries where OTC antibiotic sales remain common, they strongly influence patients’ choices of antibiotics [38]. Community pharmacists also shape patients’ decisions, particularly in settings with limited oversight of OTC antibiotic sales [39]. Understanding how and why physicians select specific antibiotics requires investigating their prescribing practices [40]. This study applies an ecological approach to assess the associations between per capita densities of primary care providers and the DIDs of “Watch” and “Reserve” group antibiotics.

An important finding is the strong positive correlation between cefpodoxime consumption and GP density, which may suggest possible overuse of this “Watch” group antibiotic. Similarly, strong positive correlations were observed between the DIDs of erythromycin, spiramycin, midecamycin, roxithromycin, josamycin, streptomycin, pefloxacin, and pipemidic acid and the densities of community internists and pediatricians, which may indicate frequent reliance on these agents in their prescription. Conversely, the strong negative associations between certain primary care provider categories and specific “Watch” and “Reserve” antibiotics may suggest the avoidance of these agents in prescribing practices. Further studies investigating prescribing behaviors among primary care providers are needed to better understand these patterns.

### 3.3. Study Limitations

The main strength of this study is the availability of a large dataset on antibiotics consumed in primary care across the entire country over the most recent eight years. Internationally, investigating antibiotic consumption in primary care sector is more challenging than in the hospital sector, and there is a paucity of such studies from LMICs. However, this study is not without limitations. One limitation is the absence of disaggregation by region within Kazakhstan, which might have provided valuable insights into regional variations in antibiotic use and allowed AMS strategies to be tailored more effectively. Another limitation is the lack of patient-level data, which could help identify individual or disease-related factors contributing to antibiotic use, such as age, as well as the absence of provider-related information that could account for urban–rural differences in service provision. In addition, the ecological design used to explore the association between antibiotic consumption and the density of primary care providers cannot capture prescribing practices with precision. Therefore, the observed correlations should be interpreted as hypothesis-generating rather than confirmatory evidence of causal links. Nevertheless, this study makes an important contribution to reshaping the national AMR framework, as it highlights gaps in current practice and provides a foundation for the development of targeted solutions.

## 4. Materials and Methods

### 4.1. Study Design

This study utilized a retrospective design and relied on two different administrative datasets. The first dataset includes information on antibiotic sales and distribution at the level of primary care, whereas the second dataset includes data on the number of healthcare providers in Kazakhstan. Overall, the study covers the period of the last eight years (2017–2024) and comprises three stages: data extraction, processing, and analysis.

### 4.2. Data Sources

The data on antibiotic consumption in primary care were obtained from Vi-ORTIS (Almaty, Kazakhstan), a company specializing in pharmaceutical market research [41]. Vi-ORTIS systematically collects and summarizes information on a wide range of pharmaceutical products sold or dispensed in Kazakhstani community pharmacies, and its data have been previously applied in pharmacoepidemiological studies on antimicrobial use [17,18,22]. The data are primarily captured via the “PharmCenter” software (https://ph.center/Account/LogOn?ReturnUrl=%2f, accessed on 22 September 2025), the most widely used pharmacy management software in Kazakhstan, which covers approximately 75% of community pharmacies. For the remaining uncovered market share, Vi-ORTIS applies proprietary modeling techniques on the basis of extrapolation from the pharmacies included in the “PharmCenter” database [41].

Data quality is ensured through multilevel verification, including monitoring products supplied to pharmacies by distributors, products sold to patients, and transactions involving pharmacy returns or transfers back to suppliers. These procedures enhance the reliability of the dataset. Access to the database is available through a subscription-based data portal. For this study, data on antibacterials for systemic use (ATC J01) were extracted for the period from 1 January 2017 to 31 December 2024 and aggregated at the ATC5 level.

Data on the number of GPs, community internists, pediatricians, and community pharmacists during the same period were retrieved from the annual statistical compendium of the MoH [42]. The demographic data required to calculate DIDs and per capita provider rates were obtained from the annual statistical reports published by the Bureau of Statistics [43].

### 4.3. Data Extraction and Processing

Data were extracted from the Vi-ORTIS portal in Excel and included the active substance, trade name, route of administration, dosage form, package size (e.g., number of tablets, sachets, bottles, or ampules per package), and number of packages sold annually. These data were then entered into the Global Antimicrobial Resistance and Use Surveillance System (GLASS-AMC) template. Developed under the WHO initiative, the GLASS-AMC methodology provides a standardized framework for calculating antibiotic consumption at the ATC5 level, expressed as defined daily doses (DDDs) per 1000 inhabitants per day [44]. The DDD values are established by the WHO Collaborating Centre for Drug Statistics Methodology, hosted by the Norwegian Institute of Public Health, and are publicly accessible [45]. For each calendar year under study, data for each ATC5 code were entered into the GLASS-AMC template in accordance with the WHO GLASS-AMC manual and data entry guidelines [46]. The GLASS-AMC template also includes built-in functions for internal data checking and validation, which are applied prior to analysis.

After validation of the data entry, the built-in functions of the GLASS-AMC template were used to calculate the DID for each ATC5 code. These calculated values were obtained for each year under study and compiled into an Excel spreadsheet. The total DID for the entire J01 category, as well as the DID for each pharmacological subgroup, were calculated via the “SUM” function in Excel. On the basis of the annual DID values, all ATC5 codes were ranked, and the top 95% of the most consumed antibiotics were identified.

Data on the number of different categories of primary care providers were also compiled in Excel, and provider density was calculated as the number of GPs, community internists, pediatricians, and community pharmacists per 1000 population, with the midyear population of Kazakhstan used as the denominator.

### 4.4. AWaRe Classification

The WHO AWaRe classification was used to categorize all J01 antibiotics into three groups—Access, Watch, and Reserve—based on their potential for resistance development. Classifications published between 2019 and 2022 [37,47] were reviewed and compared with the most recent version (2023) [48]. No antibiotics were reassigned between the Access, Watch, and Reserve groups across these updates; therefore, the 2023 classification was applied throughout. Antibiotics not included in AWaRe were categorized as Unclassified.

### 4.5. Statistical Analysis

The Statistical Package for the Social Sciences (SPSS), version 24.0, was used for time series and correlation analyses. The Average Annual Percent Change (AAPC) with its 95% CI was calculated for each ATC5 code, each pharmacological group, and for J01 overall. The “Expert Modeler” function of SPSS was applied to automatically identify the best-fitting time series model for the percentage of “Access”, “Watch”, “Reserve”, and “Unclassified” antibiotics out of total consumption, as well as to generate forecasts through 2030.

The ‘Expert Modeler’ selects the best-fit model on the basis of established criteria, including goodness-of-fit statistics, stationarity diagnostics, and predictive accuracy. ARIMA (0,1,0) was selected for the percentage of “Access” and “Watch” antibiotics, ARIMA (0,0,0) for “Reserve” antibiotics, and Simple Exponential Smoothing for “Unclassified” antibiotics. The normalized Bayesian Information Criterion (BIC) values for the forecasts were 2.912 for “Access”, 2.924 for “Watch”, −13.260 for “Reserve”, and −2.197 for “Unclassified” antibiotics. The Mean Absolute Percentage Error (MAPE) was 5.277 for “Access”, 4.839 for “Watch”, 191.338 for “Reserve”, and 20.827 for “Unclassified” antibiotics.

Pearson’s correlation coefficient was used to examine associations between the per capita density of each category of primary care provider and the consumption of “Watch” and “Reserve” group antibiotics. Strong positive or negative correlations were defined as r ≥ 0.700 or r ≤ −0.700 [49]. Statistical significance was set at *p* < 0.05.

## 5. Conclusions

The main finding of this study is that the share of “Watch” group antibiotics in the total structure of antibiotic consumption in the primary care remains substantial and has increased over the past eight years, with an AAPC of 4.49. This share is projected to increase further by 2030 if no interventions are implemented. Although the consumption of “Reserve” group antibiotics has remained low, it has also increased over the past eight years, with an AAPC of 15.35. The increasing consumption of “Watch” and “Reserve” group antibiotics contributes to the development of AMR, thereby reducing the effectiveness of available treatment options. The AMS framework of Kazakhstan needs to be strengthened to align with WHO global targets. Key strategies may include stricter enforcement of regulations to prevent OTC sales of antibiotics, revision of existing national standards of care, development of national guidelines for antibiotic prescribing, and implementation of awareness-raising campaigns targeting both the general public and primary healthcare providers.

## Figures and Tables

**Table 1:** Antibiotic consumption by pharmacological group, defined daily doses per 1000 inhabitants per day.

Pharmacological Group	Year								^0^ AAPC (95% * CI)
	2017	2018	2019	2020	2021	2022	2023	2024	
Tetracyclines	0.67	0.58	0.54	0.50	0.51	0.47	0.61	0.73	0.52 (−5.37–6.78)
Amphenicols	0.33	0.33	0.32	0.28	0.28	0.25	0.27	0.42	−0.14 (−6.64–6.81)
Penicillins	1.68	1.62	1.75	1.23	1.04	0.90	1.30	1.53	−4.55 (−12.45–4.06)
Beta-lactams	0.50	0.45	0.37	0.34	0.50	0.67	0.83	1.13	13.97 (2.77–26.40)
Cephalosporins	1.66	1.74	1.52	2.02	1.71	1.67	1.77	2.81	4.77 (−1.55–11.49)
Carbapenems	0.00	0.00	0.00	0.01	0.01	0.00	0.00	0.00	37.29 (−2.88–94.06)
Sulfonamides and their combinations	0.00	0.00	0.00	0.00	0.00	0.00	0.00	0.00	−26.61 (−71.45–88.65)
Macrolides	0.69	0.79	0.67	0.67	0.60	0.47	0.36	0.52	−8.23 (−13.65–−2.47)
Lincosamides	0.28	0.28	0.28	0.26	0.24	0.25	0.31	0.43	3.88 (−2.83–11.05)
Aminoglycosides	0.62	0.70	0.74	1.34	1.30	1.27	1.27	2.17	17.16 (9.38–25.49)
Fluoroquinolones	1.78	1.66	1.50	1.45	1.21	1.32	1.37	1.90	−1.34 (−7.26–4.97)
Quinolones	0.20	0.20	0.20	0.38	0.35	0.29	0.32	0.47	11.64 (3.48–20.46)
Combinations of antibacterials	0.08	0.08	0.03	0.02	0.03	0.02	0.03	0.03	−14.17 (−25.84–−0.67)
Glycopeptides	0.00	0.00	0.00	0.00	0.00	0.00	0.00	0.00	−4.58 (−60.16–128.53)
Polymyxins	0.00	0.00	0.00	0.00	0.00	0.00	0.00	0.00	30.75 (5.00–62.82)
Imidazoles	0.16	0.20	0.18	0.20	0.14	0.16	0.19	0.31	4.47 (−4.05–13.76)
Nitrofuran derivatives	0.61	0.58	0.51	0.44	0.46	0.47	0.47	0.81	0.78 (−7.26–9.52)
Aminocyclitols	0.15	0.13	0.15	0.13	0.13	0.12	0.07	0.16	−3.45 (−12.08–6.04)
Total	9.41	9.34	8.76	9.28	8.50	8.33	9.17	13.43	2.58 (−2.96–8.43)

^0^ AAPC—average annual percentage change. * CI—Confidence interval.

**Table 2:** Top 95% most consumed antibiotics, 2017–2024.

Substance and * AWaRe Category		Rank (%)							
		2017	2018	2019	2020	2021	2022	2023	2024
Azithromycin	Watch	6 (6.10)	4 (7.08)	4 (8.08)	1 (14.09)	1 (14.95)	1 (14.96)	1 (13.55)	1 (15.62)
Ciprofloxacin		1 (12.51)	1 (12.05)	2 (12.40)	3 (11.41)	2 (11.91)	2 (12.39)	2 (12.55)	2 (12.03)
Amoxicillin	Access	2 (10.92)	2 (11.26)	1 (14.32)	4 (10.77)	4 (8.13)	5 (7.57)	3 (11.03)	3 (8.97)
Ceftriaxone	Watch	3 (8.10)	3 (8.66)	3 (8.30)	2 (12.57)	3 (9.92)	3 (9.52)	5 (8.28)	4 (8.64)
Amoxicillin and ^0^ BLI	Access	7 (5.26)	7 (4.78)	8 (4.26)	8 (3.64)	5 (5.75)	4 (8.00)	4 (9.01)	5 (8.45)
Cefazolin		5 (6.26)	5 (6.27)	7 (4.92)	5 (5.48)	7 (4.61)	6 (4.81)	8 (3.88)	6 (5.07)
Doxycycline		8 (5.15)	9 (4.22)	5 (5.35)	6 (4.56)	6 (4.70)	7 (4.59)	6 (5.08)	8 (4.91)
Ampicillin		4 (6.36)	6 (5.10)	6 (5.03)	18 (2.13)	10 (3.84)	14 (2.82)	13 (2.86)	16 (2.14)
Cefuroxime	Watch	15 (2.21)	14 (2.37)	15 (2.51)	14 (2.30)	9 (3.85)	8 (3.82)	7 (4.28)	7 (4.92)
Levofloxacin		16 (2.15)	16 (2.17)	16 (2.21)	7 (4.04)	8 (4.11)	9 (3.46)	9 (3.5)	10 (3.49)
Nitrofurantoin	Access	12 (3.50)	12 (3.27)	11 (3.12)	15 (2.23)	11 (3.43)	10 (3.41)	11 (2.94)	9 (3.54)
Sulfamethoxazole and ^α^ TMP		10 (4.05)	8 (4.33)	10 (3.41)	9 (3.38)	13 (3.19)	11 (3.16)	14 (2.75)	13 (2.69)
Chloramphenicol		11 (3.54)	11 (3.49)	9 (3.62)	10 (3.4)	12 (3.33)	12 (2.97)	12 (2.92)	12 (3.09)
Clarithromycin	Watch	14 (2.46)	15 (2.36)	14 (2.63)	13 (2.36)	14 (2.75)	13 (2.94)	10 (3.41)	11 (3.23)
Furazidin	Access	13 (3.01)	13 (2.93)	13 (2.70)	12 (2.48)	16 (1.96)	15 (2.19)	15 (2.20)	14 (2.46)
Gentamicin		9 (4.30)	10 (3.94)	12 (3.06)	11 (2.55)	26 (0.31)	23 (0.65)	19 (0.87)	40 (0.01)
Metronidazole		18 (1.71)	17 (2.11)	18 (2.04)	16 (2.15)	17 (1.67)	16 (1.92)	16 (2.08)	15 (2.31)
Tetracycline		17 (1.95)	18 (1.95)	21 (0.85)	21 (0.87)	18 (1.33)	18 (1.05)	17 (1.56)	22 (0.09)
Nitroxoline	^∞^ UC	19 (1.28)	20 (1.13)	19 (1.39)	19 (1.20)	19 (1.18)	20 (1.04)	21 (0.64)	19 (0.75)
Norfloxacin	Watch	20 (1.09)	22 (0.91)	20 (0.90)	20 (0.87)	20 (1.12)	19 (1.05)	23 (0.61)	18 (1.03)
Cefixime		27 (0.57)	23 (0.86)	23 (0.84)	25 (0.55)	21 (0.70)	21 (1.02)	18 (1.36)	17 (1.36)
Ofloxacin		22 (0.84)	25 (0.73)	25 (0.60)	23 (0.62)	22 (0.69)	22 (0.69)	20 (0.74)	20 (0.71)
Erythromycin		21 (1.04)	21 (1.00)	22 (0.85)	24 (0.55)	25 (0.47)	25 (0.40)	26 (0.35)	25 (0.07)

* AWaRe—Access, Watch, Reserve Classification. ^0^ BLI—Beta-Lactamase Inhibitor. ^α^ TMP—Trimethoprim. ^∞^ UC—unclassified.

**Table 3:** Projected rates of antibiotic consumption by AWaRe categories through 2030.

Year	“Access” Group Antibiotics, % (95% CI)	“Watch” Group Antibiotics, % (95% CI)	“Reserve” Group Antibiotics, % (95% CI)	“Unclassified” Group Antibiotics, % (95% CI)
2025	42.91 (33.78–52.05)	56.25 (47.06–65.44)	0.001 (−0.001–0.004)	0.89 (0.20–1.58)
2026	41.07 (28.16–53.99)	58.15 (45.15–71.14)	0.001 (−0.001–0.004)	0.89 (−0.09–1.87)
2027	39.23 (23.41–55.05)	60.04 (44.13–75.96)	0.001 (−0.001–0.004)	0.89 (−0.31–2.09)
2028	37.39 (19.13–55.66)	61.94 (43.56–80.32)	0.001 (−0.001–0.004)	0.89 (−0.50–2.27)
2029	35.55 (15.13–55.98)	63.84 (43.29–84.38)	0.001 (−0.001–0.004)	0.89 (−0.66–2.44)
2030	33.71 (11.34–56.09)	65.73 (43.22–88.24)	0.001 (−0.001–0.004)	0.89 (−0.81–2.58)
Model parameters	ARIMA (0.1.0) *p* = 0.240	ARIMA (0.1.0) *p* = 0.230	ARIMA (0.0.0) *p* = 0.011	Simple *p* = 0.028

**Table 4:** Correlations between the consumption of “Watch” and “Reserve” group antibiotics and different categories of primary care professionals, 2017–2024.

Substance (^0^ ATC5 Code)	Primary Care Professionals					
	* GPs		Internists and Pediatricians		Pharmacists	
	r	*p* Value	r	*p* Value	r	*p* Value
Cefpodoxime (J01DD13)	0.941	<0.001	−0.934	0.001	-	-
Ceftaroline-fosamil (J01DI02)	-	-	-	-	−0.740	0.036
Erythromycin (J01FA01)	−0.911	0.002	0.967	<0.001	-	-
Spiramycin (J01FA02)			0.703	0.052		
Midecamycin (J01FA03)	-	-	0.807	0.015	-	-
Roxithromycin (J01FA06)	−0.841	0.009	0.901	0.002		
Josamycin (J01FA07)	-	-	0.792	0.019	-	-
Streptomycin (J01GA01)	−0.723	0.043	0.726	0.041	-	-
Kanamycin (J01GB04)	−0.711	0.048			-	-
Pefloxacin (J01MA03)	−0.929	0.001	0.928	0.002	-	-
Moxifloxacin (J01MA14)	0.888	0.003	−0.881	0.004	-	-
Pipemidic acid (J01MB04)	−0.944	<0.001	0.912	0.002	-	-
Vancomycin (J01XA01)	-	-	-	-	−0.766	0.027
Colistin (J01XB01)			−0.700	0.053		

* GPs—General practitioners. ^0^ ATC5—Anatomic therapeutic classification, level 5.

## Data Availability

The data presented in this study are provided as Supplementary Materials.

## Supplementary Materials

The following supporting information can be downloaded at: https://www.mdpi.com/article/10.3390/antibiotics14100963/s1, Table S1: Antibiotic consumption in primary care, 2017–2024.

## Abbreviations

The following abbreviations are used in this manuscript:

AAPS	Average Annual Percent Change
AMR	Antimicrobial Resistance
AMS	Antimicrobial Stewardship
ATC	Anatomical Therapeutic Chemical Classification
AWaRe	Access, Watch, Reserve
DDD	Defined Daily Doses
DID	Defined Daily Doses per 1000 Inhabitants per Day
GLASS-AMC	Global Antimicrobial Resistance and Use Surveillance System
GP	General Practitioner
LMICs	Low- and Middle-Income Countries
MoH	Ministry of Health
OTC	Over-the-Counter
SPSS	Statistical Package for the Social Sciences
WHO	World Health Organization
95% CI	95% Confidence Interval
