# Supplementary material for: Trends in ‘Watch’ and ‘Reserve’ Antibiotic Use in Primary Care in Kazakhstan: The Imperative for Enhancing Stewardship Strategies
Source: Antibiotics (Basel). 2025 Sep 25;14(10):963. doi: 10.3390/antibiotics14100963 (PMC12561045; doi:10.3390/antibiotics14100963)
Supplement: Supplementary file 1 [file antibiotics-14-00963-s001.zip › antibiotics-3881281-supplementary.pdf]

**Table S1.** Antibiotic consumption in primary care, 2017-2024.

| Substance (ΩATC5 code) | Pharmacological subgroup (ATC3 code) | AWaRe∞ category | DIDα 2017 | 2018 | 2019 | 2020 | 2021 | 2022 | 2023 | 2024 | AAPC⁰ (95% CI*) |
|---|---|---|---|---|---|---|---|---|---|---|---|
| Doxycycline (J01AA02) | Tetracyclines (J01A) | Access | 0.48 | 0.39 | 0.47 | 0.42 | 0.40 | 0.38 | 0.47 | 0.66 | 2.79 (-3.79; 9.81) |
| Tetracycline (J01AA07) | | | 0.18 | 0.18 | 0.07 | 0.08 | 0.11 | 0.09 | 0.14 | 0.07 | -8.39 (-20.17; 5.13) |
| Tigecycline (J01AA12) | | Reserve | 0.00 | 0.00 | 0.00 | 0.00 | 0.00 | 0.00 | 0.00 | 0.00 | - |
| Chloramphenicol (J01BA01) | Amphenicols (J01B) | Access | 0.33 | 0.33 | 0.32 | 0.28 | 0.28 | 0.25 | 0.27 | 0.41 | -0.27 (-6.60; 6.48) |
| Thiamphenicol (J01BA02) | | | 0.00 | 0.00 | 0.00 | 0.00 | 0.00 | 0.00 | 0.00 | 0.01 | - |
| Ampicillin (J01CA01) | Beta-lactam antibacterials, penicillins (J01C) | | 0.60 | 0.48 | 0.44 | 0.20 | 0.33 | 0.23 | 0.26 | 0.29 | -10.74 (-19.79; -0.67) |
| Amoxicillin (J01CA04) | | | 1.03 | 1.05 | 1.26 | 1.00 | 0.69 | 0.63 | 1.01 | 1.20 | -1.82 (-11.09; 8.41) |
| Benzylpenicillin (J01CE01) | | | 0.06 | 0.04 | 0.03 | 0.02 | 0.02 | 0.03 | 0.02 | 0.03 | -9.77 (-19.22; 0.79) |
| Benzathine Benzylpenicillin (J01CE08) | | | 0.00 | 0.01 | 0.00 | 0.00 | 0.00 | 0.00 | 0.00 | 0.00 | - |
| Combinations of penicillins (J01CE30) | | UnclassifiedB | 0.00 | 0.04 | 0.01 | 0.01 | 0.00 | 0.01 | 0.01 | 0.01 | -14.74 (-38.81; 18.80) |
| Ampicillin and beta-lactamase inhibitor (J01CR01) | | Access | 0.00 | 0.00 | 0.00 | 0.00 | 0.00 | 0.00 | 0.00 | 0.00 | - |
| Amoxicillin and beta-lactamase inhibitor (J01CR02) | | | 0.50 | 0.45 | 0.37 | 0.34 | 0.49 | 0.67 | 0.83 | 1.13 | 13.91 (2.61; 26.45) |
| Sultamicillin (J01CR04) | | | 0.00 | 0.00 | 0.00 | 0.00 | 0.00 | 0.00 | 0.00 | 0.00 | - |
| Piperacillin and beta-lactamase inhibitor (J01CR05) | | Watch | 0.00 | 0.00 | 0.00 | 0.00 | 0.00 | 0.00 | 0.00 | 0.00 | 3.63 (-38.59; 74.87) |
| Combinations of penicillins (J01CR50) | | Unclassified | 0.00 | 0.00 | 0.00 | 0.00 | 0.01 | 0.00 | 0.00 | 0.00 | - |
| Cefalexin (J01DB01) | Other beta-lactam antibacterials (J01D) | Access | 0.00 | 0.00 | 0.00 | 0.00 | 0.00 | 0.00 | 0.00 | 0.00 | - |
| Cefazolin (J01DB04) | | | 0.59 | 0.59 | 0.43 | 0.51 | 0.39 | 0.40 | 0.36 | 0.68 | -2.32 (-10.93; 7.11) |
| Cefuroxime (J01DC02) | | Watch | 0.21 | 0.22 | 0.22 | 0.21 | 0.33 | 0.32 | 0.39 | 0.66 | 15.99 (8.00; 24.58) |
| Cefprozil (J01DC10) | | | 0.02 | 0.02 | 0.02 | 0.01 | 0.01 | 0.01 | 0.02 | 0.02 | 3.38 (-6.61; 14.45) |
| Cefotaxime (J01DD01) | | | 0.00 | 0.00 | 0.00 | 0.00 | 0.00 | 0.00 | 0.00 | 0.00 | - |
| Ceftazidime (J01DD02) | | | 0.01 | 0.01 | 0.01 | 0.01 | 0.01 | 0.01 | 0.01 | 0.01 | 2.06 (-4.80; 9.40) |

| | | | | | | | | | | | |
|---|---|---|---|---|---|---|---|---|---|---|---|
| Ceftriaxone (J01DD04) | | | 0.76 | 0.81 | 0.73 | 1.17 | 0.84 | 0.77 | 0.76 | 1.16 | 2.95 (-4.26; 10.70) |
| Cefixime (J01DD08) | | | 0.05 | 0.08 | 0.07 | 0.05 | 0.06 | 0.08 | 0.12 | 0.18 | 14.42 (1.81; 28.58) |
| Cefoperazone (J01DD12) | | | 0.00 | 0.00 | 0.00 | 0.00 | 0.00 | 0.00 | 0.00 | 0.00 | -42.44 (-63.71; -8.72) |
| Cefpodoxime (J01DD13) | | | 0.01 | 0.02 | 0.03 | 0.04 | 0.05 | 0.05 | 0.05 | 0.03 | 19.38 (3.56; 37.61) |
| Cefdinir (J01DD15) | | | 0.00 | 0.00 | 0.01 | 0.01 | 0.01 | 0.02 | 0.06 | 0.06 | 67.90 (41.08; 99.82) |
| Ceftazidime and beta-lactamase inhibitor (J01DD52) | | Reserve | 0.00 | 0.00 | 0.00 | 0.00 | 0.00 | 0.00 | 0.00 | 0.00 | - |
| Cefoperazone and beta-lactamase inhibitor (J01DD62) | | Unclassified | 0.00 | 0.00 | 0.00 | 0.00 | 0.00 | 0.00 | 0.00 | 0.00 | 72.24 (43.35; 106.96) |
| Cefepime (J01DE01) | Other beta-lactam antibacterials (J01D) | | 0.00 | 0.00 | 0.00 | 0.00 | 0.00 | 0.01 | 0.00 | 0.00 | 37.29 (-6.95; 102.57) |
| Meropenem (J01DH02) | | | 0.00 | 0.00 | 0.00 | 0.01 | 0.01 | 0.00 | 0.00 | 0.00 | 40.49 (-1.14; 99.67) |
| Ertapenem (J01DH03) | | | 0.00 | 0.00 | 0.00 | 0.00 | 0.00 | 0.00 | 0.00 | 0.00 | -13.94 (-58.35; 77.84) |
| Doripenem (J01DH04) | | | 0.00 | 0.00 | 0.00 | 0.00 | 0.00 | 0.00 | 0.00 | 0.00 | -42.02 (-72.08; 20.39) |
| Imipenem and cilastatin (J01DH51) | | Watch | 0.00 | 0.00 | 0.00 | 0.00 | 0.00 | 0.00 | 0.00 | 0.00 | - |
| Ceftaroline fosamil (J01DI02) | | Reserve | 0.00 | 0.00 | 0.00 | 0.00 | 0.00 | 0.00 | 0.00 | 0.00 | - |
| Sulfaisodimidine (J01EB01) | Sulfonamides and trimethoprim (J01E) | Access | 0.07 | 0.17 | 0.18 | 0.20 | 0.20 | 0.12 | 0.00 | 0.00 | - |
| Sulfamethoxazole and trimethoprim (J01EE01) | | | 0.38 | 0.40 | 0.30 | 0.31 | 0.27 | 0.26 | 0.25 | 0.36 | -3.83 (-9.60; 2.31) |
| Erythromycin (J01FA01) | Macrolides, lincosamides, and streptogramins (J01F) | Watch | 0.10 | 0.09 | 0.07 | 0.05 | 0.04 | 0.03 | 0.03 | 0.06 | -13.06 (-22.16; -2.90) |
| Spiramycin (J01FA02) | | | 0.07 | 0.07 | 0.07 | 0.06 | 0.05 | 0.03 | 0.05 | 0.08 | -4.47 (-15.66; 8.20) |
| Midecamycin (J01FA03) | | | 0.05 | 0.03 | 0.03 | 0.03 | 0.02 | 0.02 | 0.01 | 0.01 | -17.43 (-24.11;-10.16) |
| Roxithromycin (J01FA06) | | | 0.03 | 0.02 | 0.02 | 0.01 | 0.01 | 0.01 | 0.01 | 0.01 | -15.63 (-20.36;-10.62) |
| Josamycin (J01FA07) | | | 0.05 | 0.06 | 0.05 | 0.04 | 0.00 | 0.00 | 0.00 | 0.00 | -68.26 (-83.32;-39.60) |
| Clarithromycin (J01FA09) | | | 0.23 | 0.22 | 0.23 | 0.22 | 0.23 | 0.25 | 0.31 | 0.43 | 7.88 (1.59; 14.55) |

| | | | | | | | | | | |
|---|---|---|---|---|---|---|---|---|---|---|
| Azithromycin (J01FA10) | | | 0.57 | 0.66 | 0.71 | 1.31 | 1.27 | 1.25 | 1.24 | 2.10 | 17.97 (9.98; 26.53) |
| Clindamycin (J01FF01) | | Access | 0.00 | 0.00 | 0.00 | 0.00 | 0.00 | 0.00 | 0.00 | 0.00 | 8.94 (0.77; 17.77) |
| Lincomycin (J01FF02) | | | 0.03 | 0.02 | 0.02 | 0.02 | 0.02 | 0.02 | 0.02 | 0.06 | 2.14 (-13.20; 20.18) |
| Streptomycin (J01GA01) | | Watch | 0.01 | 0.02 | 0.01 | 0.01 | 0.01 | 0.00 | 0.01 | 0.01 | -6.01 (-23.96; 16.18) |
| Tobramycin (J01GB01) | Aminoglycoside antibacterials (J01G) | | 0.00 | 0.00 | 0.00 | 0.00 | 0.00 | 0.00 | 0.00 | 0.00 | - |
| Gentamicin (J01GB03) | | Access | 0.40 | 0.37 | 0.27 | 0.24 | 0.03 | 0.05 | 0.08 | 0.00 | -42.29 (-58.36;-20.02) |
| Kanamycin (J01GB04) | | Watch | 0.00 | 0.00 | 0.00 | 0.00 | 0.00 | 0.00 | 0.00 | 0.00 | - |
| Amikacin (J01GB06) | | Access | 0.01 | 0.01 | 0.01 | 0.01 | 0.01 | 0.01 | 0.01 | 0.04 | 12.14 (-3.93; 30.89) |
| Ofloxacin (J01MA01) | | | 0.08 | 0.07 | 0.05 | 0.06 | 0.06 | 0.06 | 0.07 | 0.10 | 1.87 (-5.67; 10.03) |
| Ciprofloxacin (J01MA02) | | | 1.18 | 1.13 | 1.09 | 1.06 | 1.01 | 1.11 | 1.15 | 1.62 | 2.81 (-2.34; 8.22) |
| Pefloxacin (J01MA03) | | | 0.01 | 0.01 | 0.00 | 0.00 | 0.00 | 0.00 | 0.00 | 0.00 | - |
| Norfloxacin (J01MA06) | | Watch | 0.10 | 0.08 | 0.08 | 0.08 | 0.10 | 0.09 | 0.06 | 0.14 | 0.56 (-9.47; 11.70) |
| Lomefloxacin (J01MA07) | Quinolone antibacterials (J01M) | | 0.00 | 0.00 | 0.00 | 0.00 | 0.00 | 0.00 | 0.00 | 0.00 | 5.10 (-9.57; 22.15) |
| Levofloxacin (J01MA12) | | | 0.20 | 0.20 | 0.19 | 0.38 | 0.35 | 0.29 | 0.32 | 0.47 | 11.69 (3.45; 20.58) |
| Moxifloxacin (J01MA14) | | | 0.01 | 0.01 | 0.02 | 0.02 | 0.03 | 0.02 | 0.03 | 0.03 | 22.92 (9.74; 37.69) |
| Nalidixic acid (J01MB02) | | | 0.00 | 0.00 | 0.00 | 0.00 | 0.00 | 0.00 | 0.00 | 0.00 | - |
| Pipemidic acid (J01MB04) | | | 0.07 | 0.07 | 0.01 | 0.00 | 0.00 | 0.00 | 0.00 | 0.00 | -93.12 (-99.00;-52.60) |
| Ofloxacin and ornidazole (J01RA09) | | Unclassified | 0.00 | 0.00 | 0.02 | 0.02 | 0.00 | 0.00 | 0.00 | 0.00 | - |
| Ciprofloxacin and metronidazole (J01RA10) | Combinations of antibacterials (J01R) | | 0.00 | 0.00 | 0.00 | 0.00 | 0.00 | 0.00 | 0.00 | 0.00 | - |
| Ciprofloxacin and ornidazole (J01RA12) | | | 0.00 | 0.00 | 0.01 | 0.00 | 0.00 | 0.00 | 0.00 | 0.00 | - |
| Vancomycin (J01XA01) | | Watch | 0.00 | 0.00 | 0.00 | 0.00 | 0.00 | 0.00 | 0.00 | 0.00 | -4.01 (-59.69; 128.58) |
| Colistin (J01XB01) | | Reserve | 0.00 | 0.00 | 0.00 | 0.00 | 0.00 | 0.00 | 0.00 | 0.00 | 30.62 (4.92; 62.60) |
| Polymyxin B (J01XB02) | | | 0.00 | 0.00 | 0.00 | 0.00 | 0.00 | 0.00 | 0.00 | 0.00 | - |
| Metronidazole (J01XD01) | Other antibacterials (J01X) | | 0.16 | 0.20 | 0.18 | 0.20 | 0.14 | 0.16 | 0.19 | 0.31 | 4.60 (-3.93; 13.90) |
| Tinidazole (J01XD02) | | | 0.00 | 0.00 | 0.00 | 0.00 | 0.00 | 0.00 | 0.00 | 0.00 | - |
| Ornidazole (J01XD03) | | Access | 0.00 | 0.00 | 0.00 | 0.00 | 0.00 | 0.00 | 0.00 | 0.00 | - |
| Nitrofurantoin (J01XE01) | | | 0.33 | 0.31 | 0.27 | 0.21 | 0.29 | 0.28 | 0.27 | 0.48 | 2.89 (-6.04; 12.67) |

| | | | | | | | | | | |
|---|---|---|---|---|---|---|---|---|---|---|
| Furazidin (J01XE03) | | 0.28 | 0.27 | 0.24 | 0.23 | 0.17 | 0.18 | 0.20 | 0.33 | -1.86 (-10.61; 7.74) |
| Fosfomycin (J01XX01) | Watch | 0.02 | 0.02 | 0.02 | 0.02 | 0.03 | 0.03 | 0.01 | 0.06 | 11.34 (-4.72; 30.12) |
| Spectinomycin (J01XX04) | Access | 0.01 | 0.01 | 0.01 | 0.00 | 0.00 | 0.00 | 0.00 | 0.00 | - |
| Nitroxoline (J01XX07) | Unclassified | 0.12 | 0.11 | 0.12 | 0.11 | 0.10 | 0.09 | 0.06 | 0.10 | -6.19 (-12.76; 0.88) |
| Linezolid (J01XX08) | Reserve | 0.00 | 0.00 | 0.00 | 0.00 | 0.00 | 0.00 | 0.00 | 0.00 | 6.56 (-29.45; 60.97) |
| Total DID** | | 9.41 | 9.34 | 9.41 | 9.34 | 8.79 | 9.30 | 8.50 | 8.33 | 9.17 |

ΩATC5 - Anatomical Therapeutic Chemical classification

∞AWaRe – Access, Watch, Reserve Classification.

αDID – Defined Daily Doses per 1000 inhabitants per day.

⁰AAPC – Average Annual Percent Change.

*CI – Confidence Interval
